# Transient receptor potential ankyrin 1 promoter methylation and peripheral pain sensitivity in Crohn’s disease

**DOI:** 10.1186/s13148-019-0796-9

**Published:** 2019-12-31

**Authors:** Sara Gombert, Mathias Rhein, Andreas Winterpacht, Tino Münster, Thomas Hillemacher, Andreas Leffler, Helge Frieling

**Affiliations:** 1 Laboratory for Molecular Neuroscience, Department of Psychiatry, Socialpsychiatry and Psychotherapy, Hannover Medical School, Feodor-Lynen-Str. 35, 30625 Hannover, Germany; 2 Institute of Human Genetics, Friedrich-Alexander University Erlangen-Nuremberg, Erlangen, Germany; 3 Department of Anesthesiology, Friedrich-Alexander University Erlangen-Nuremberg, Erlangen, Germany; 4 Clinic for Anesthesiology and Critical Care, Hospital of the Order of St.John of God Regensburg, Regensburg, Germany; 5 Department of Psychiatry and Psychotherapy, Paracelsus Medical University Nuremberg, Nuremberg, Germany; 6 Department of Anesthesiology and Intensive Care Medicine, Hannover Medical School, Hannover, Germany

**Keywords:** *TRPA1*, Promoter methylation, Pressure pain, IBD, Crohn’s disease

## Abstract

**Background:**

Crohn’s disease is a chronic inflammatory disorder of the gastrointestinal tract associated with abdominal pain and diarrhea. Pain caused by Crohn’s disease likely involves neurogenic inflammation which seems to involve the ion channel transient receptor potential ankyrin 1 (TRPA1). Since the promoter methylation of *TRPA1* was shown to influence pain sensitivity, we asked if the expression of TRPA1 is dysregulated in patients suffering from Crohn’s disease.

The methylation rates of CpG dinucleotides in the *TRPA1* promoter region were determined from DNA derived from whole blood samples of Crohn patients and healthy participants. Quantitative sensory testing was used to examine pain sensitivities.

**Results:**

Pressure pain thresholds were lower in Crohn patients as compared to healthy participants, and they were also lower in females than in males. They correlated inversely with the methylation rate at the CpG − 628 site of the *TRPA1* promoter. This effect was more pronounced in female compared to male Crohn patients. Similar results were found for mechanical pain thresholds. Furthermore, age-dependent effects were detected. Whereas the CpG − 628 methylation rate declined with age in healthy participants, the methylation rate in Crohn patients increased. Pressure pain thresholds increased with age in both cohorts.

**Conclusions:**

The *TRPA1* promoter methylation appears to be dysregulated in patients suffering from Crohn’s disease, and this effect is most obvious when taking gender and age into account. As *TRPA1* is regarded to be involved in pain caused by neurogenic inflammation, its aberrant expression may contribute to typical symptoms of Crohn’s disease.

## Background

Inflammatory bowel diseases (IBD) including Crohn’s disease and ulcerative colitis are chronic inflammatory disorders of the gastrointestinal tract resulting from multiple factors that seem to trigger an abnormal activation of the mucosal immune system through commensal microflora. Studies employing animal models on IBD reported that sensory neurons innervating the gastrointestinal tract are likely to be involved in these disorders [1–10]. Activation of sensory neurons results in a release of neuropeptides, including calcitonin gene-related peptide (CGRP) and substance P (SP). Both these peptides induce several effects including vasodilation, an autocrine sensitization of sensory neurons, extravasation and activation of immune cells [6]. Thus, neuropeptide-induced effects seem to very well correlate with the clinical symptoms of IBD. Certain members of the transient receptor potential (TRP) ion channels, which are expressed in sensory neurons, have been shown to contribute to IBD-like symptoms [1, 3, 4] or colonic distension pain [11] in rodent models. TRP channels are activated by a wide range of chemical compounds, among them, some possess mechano- or thermosensitivity [12]. Substances, which are associated with TRPA1 activation, are isothiocyanates from mustard oil, wasabi, and horseradish, Δ(9)-tetrahydrocannabinol from marijuana, allicin from garlic, cinnamaldehyde from cinnamon oil, as well as reactive oxygen species and others [13–16]. TRPA1 possesses a species-dependent thermosensitivity [17–20], and a recent study suggests that, at least in humans, the channel is able to respond to both cold and heat [21]. Furthermore, in mice, TRPA1 was shown to be mechanosensitive [22–27], which correlates with studies on human volunteers showing that TRPA1 agonists are able to induce mechanical hypersensitivity when applied on the skin [28]. The TRPA1 agonist TNBS (2,4,6-trinitrobenzene-sulfonic-acid), used for induction of colitis, caused IBD-like symptoms in mice due to a release of SP and CGRP [3]. Also in patients suffering from IBD, an increase in SP was observed [29]. Similarly, previous studies demonstrated that the TRPA1-agonists isothiocyanate and formalin also induce colitis in rodents [30, 31]. Although wild-type mice do not spontaneously develop intestinal inflammation as humans do [32] and the available animal models of IBD do not fully resemble the human condition [33], the symptoms and morphology induced by the TRPA1 agonists coincide very well with human IBD. Given the fact that there are several frequently used models based on TRPA1 agonists causing an IBD-like morphology in mice, this ion channel could be involved in the development of IBD in humans as well. In human colon samples from patients suffering from ulcerative colitis or Crohn’s disease, TRPA1 was found to be strongly upregulated [34], suggesting dysregulation of TRPA1-expression to possibly contribute to the development, maintenance, or typical symptoms of IBD.

TRPA1 was demonstrated to mediate pain-like behavior in rodent models of visceral pain, and also visceral hyperalgesia developing in models of IBD was reported to be mediated by TRPA1 [30, 35–38]. There is little doubt that TRPA1 is an important determinant of pain sensitivity in humans, as well. A rare hereditary gain-of-function mutation is associated with an intensive pain phenotype [39], and a SNP (single-nucleotide polymorphism) in the *TRPA1* gene was reported to be associated with paradoxical heat sensations in neuropathic pain patients [40]. Here, we refer to the relevance of the epigenetic regulation of *TRPA1*. The promoter methylation of *TRPA1* was initially reported to be strongly associated with thermal pain thresholds [41]. We and others could demonstrate that the methylation rate of the *TRPA1* promoter also seems to predict pressure pain thresholds in healthy individuals as well as neuropathic pain symptoms in patients suffering from chronic pain [42, 43]. Hypothesizing that the previously reported elevated expression of TRPA1 in individuals suffering from Crohn’s disease might be due to epigenetic regulation, we used DNA from whole blood of Crohn patients and healthy participants to analyze the methylation status of individual CpG sites in the *TRPA1* promoter with regard to pain sensitivity.

## Results

### Quantitative sensory testing and postoperative morphine consumption in Crohn patients

Mean values for QST parameters and postoperative morphine consumption of female and male Crohn patients are shown in Table 1. Deviance from normal distribution was checked according to Shapiro-Wilk. HPT was shown to be the only normally distributed variable of the QST data (*P* = 0.052). Comparison between female and male Crohn patients by calculation with Mann-Whitney *U* test (random sample selection) revealed only significant differences for HPT (*P* = 0.020) and PPT (*P* = 0.039), with females showing lower thresholds as compared to males.

**Table 1:** Patient-controlled analgesia and quantitative sensory testing in Crohn patients

	Female	Male	*p* value
PCA (morphine mg/kg/h)	0.05 ± 0.03	0.04 ± 0.02	0.489
CDT (°C, difference from 32 °C baseline)	− 1.6 ± 1.1	− 1.6 ± 1.6	0.479
WDT (°C, difference from 32 °C baseline)	2.1 ± 1.2	2.9 ± 2.1	0.337
CPT (°C, difference from 32 °C baseline)	− 17.1 ± 7.8	− 15.1 ± 8.7	0.515
HPT (°C, difference from 32 °C baseline)	41.4 ± 3.4	43.9 ± 4.1	0.020*
MDT (mN)	1.6 ± 4.5	1.0 ± 1.0	0.399
MPT (mN)	261.7 ± 236.5	231.4 ± 188.5	0.913
MPS (rating 0–100)	0.8 ± 0.9	0.9 ± 1.1	0.808
WUR (ratio)	3.0 ± 2.4	2.7 ± 2.8	0.483
VDT (x/8)	7.7 ± 0.4	7.4 ± 0.7	0.243
PPT (N)	40.6 ± 17.9	53.9 ± 22.9	0.039*

All data are presented as mean ± SD.

*PCA* patient-controlled analgesia, *CDT* cold detection threshold, *WDT* warm detection threshold, *CPT* cold pain threshold, *HPT* heat pain threshold, *MDT* mechanical detection threshold, *MPT* mechanical pain threshold, *MPS* mechanical pain sensitivity, *WUR* wind-up ratio, *VDT* vibration detection threshold, *PPT* pressure pain threshold

*p < 0.05

### Comparison of the pressure pain thresholds in healthy participants as compared to Crohn patients

**Fig. 1:**
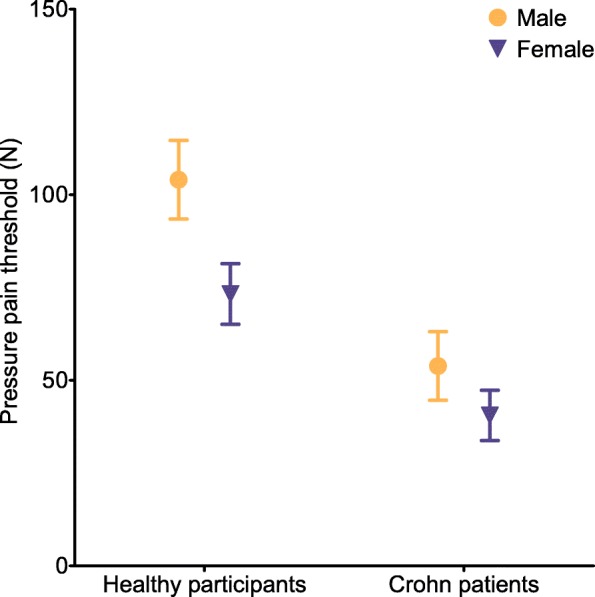
Pressure pain threshold of healthy participants and Crohn patients. PPT varies significantly between cohorts and is lower for females than for males. Error bars are presented as 95% CI. Significant differences between cohorts (Mann-Whitney *U*, *p* < 0.001), significant differences between genders within cohorts (Mann-Whitney *U*, healthy participants *p* < 0.001, Crohn patients *p* = 0.039)

The measurement of PPT via algometer revealed significant differences between healthy participants and Crohn patients (Mann-Whitney *U*, *p* < 0.001, Fig. 1). Healthy participants, as well as Crohn patients, showed significant differences between female and male in regard to the PPT (Mann-Whitney *U*, healthy participants: *p* < 0.001, Crohn patients *p* = 0.039). The mean PPT in healthy subjects was 73.2 N for females and 104.1 N for males, and the corresponding values in Crohn patients were significantly lower with 40.6 N for females and 53.9 N for males.

### Association between CpG − 628 methylation rate and pressure pain threshold in healthy participants and Crohn patients

As separate analyses of the single CpGs and QST, as well as PCA parameter interactions, led to inconclusive results, we decided to perform a more in-depth analysis only of CpG − 628 methylation with respect to PPT in Crohn patients, since previous studies showed a superordinate role for this specific CpG site on pain thresholds of healthy individuals [41, 42].

**Fig. 2:**
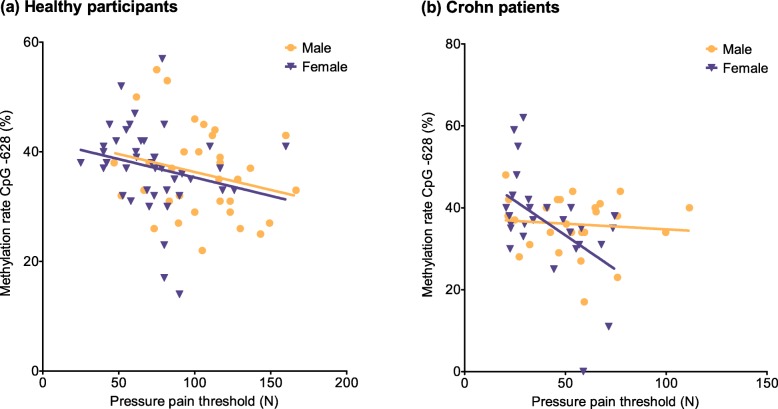
Correlation between CpG − 628 methylation and pressure pain threshold. PPT is lower with higher methylation rates. **a** Healthy participants; linear regression shows no difference between genders (males *R*^2^ = 0.057, females *R*^2^ = 0.050). **b** Crohn patients; linear regression shows large differences between genders (males *R*^2^ = 0.008, females *R*^2^ = 0.250)

Unfortunately, PPT was the only QST parameter measured in both cohorts, enabling a comparison between healthy subjects and Crohn patients. The linear association between CpG − 628 methylation and PPT is illustrated in Fig. 2. Scatter plots for healthy participants and Crohn patients are separated into genders. The higher the CpG − 628 methylation ratio, the lower is the PPT in both cohorts. Whereas the calculation of the linear regression reveals similar results between healthy males (*R*^2^ = 0.057) and females (*R*^2^ = 0.050), the corresponding linear regression for Crohn patients reveals a strong predictive potential for female (*R*^2^ = 0.250) as compared to male patients (*R*^2^ = 0.008) as well as to the healthy volunteer group.

### Association between CpG − 628 methylation rate and other QST, PCA, demographic, and disease-associated parameters in Crohn patients

**Fig. 3:**
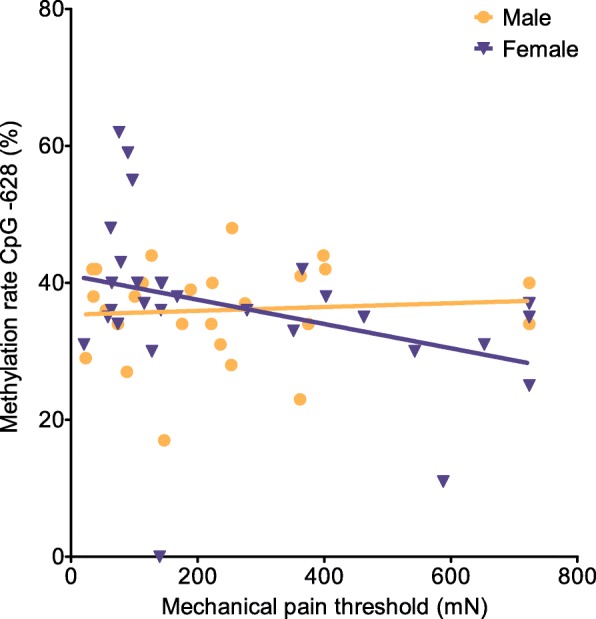
Association between CpG − 628 methylation rate and the variable mechanical pain threshold in Crohn patients. In female patients, MPT is lower with higher methylation rates. In male patients, it is inappreciably higher (linear regression; males *R*^2^ = 0.006, females *R*^2^ = 0.122)

We next calculated a stepwise multiple linear regression of Crohn patient data using the BACKWARD method. We included age, gender, and CpG − 628 methylation as predictors and separately for each analysis values for PCA and QST (Table 1) as well as demographic and disease-associated data (Table 2) as the dependent variable. We found CpG − 628 to be a significant predictor for the dependent variables PPT, MPT, and BMI. Scatter plots for PPT and MPT are shown in Figs. 2 and 3. Pain thresholds are lower with higher methylation rates, and this effect is stronger pronounced in female as compared to male patients. The best-fitting model for PPT (*R*^2^ = 0.187, *R*^2^_corr_ = 0.155, *F*_(2)_ = 5.970, *p* = 0.005) included gender and CpG − 628 methylation as significant predictors (gender β = 0.308, *T* = 2.464, *p* = 0.017, CpG − 628 methylation β = − 0.296, *T* = − 2.365, *p* = 0.022). In case of MPT, the best fitting model (*R*^2^ = 0.100, *R*^2^_corr_ = 0.066, *F*_(2)_ = 2.901, *p* = 0.064) included only CpG − 628 methylation as significant predictor (β = − 0.286, *T* = − 2.089, *p* = 0.042). Calculation of the linear regression of the MPT reveals differences between female (*R*^2^ = 0.122) and male patients (*R*^2^ = 0.006). The predictor age did not reach significance level (β = 0.238, *T* = 1.734, *P* = 0.089). To analyze if the observed effect of BMI is gender-related, we calculated a regression separately for both genders, including age, CpG − 628 methylation and BMI as predictors and PPT as the dependent variable. CpG − 628 methylation in female subjects was found to be the only significant predictor for PPT (β = − 0.533, *T* = − 2.722, *P* = 0.012). When calculating a stepwise multiple linear regression (using the BACKWARD method) with the same predictors and dependent variable, the best fitting model for female patients for PPT (*R*^2^ = 0.312, *R*^2^_corr_ = 0.257, *F*_(2)_ = 5.676, *p* = 0.009) included only CpG − 628 methylation as significant predictor (β = − 0.561, *T* = − 3.249, *p* = 0.003). Again, the predictor age did not reach the significance level (β = 0.305, *T* = 1.763, *P* = 0.090). For male patients, none of the predictors was significant. Therefore, BMI was not considered a relevant factor, but rather a gender-related side effect.

**Table 2:** Demographic and disease-associated parameters for Crohn patients and healthy volunteers

Collective	Crohn patients (*n* = 55)		Healthy volunteers (*n* = 75)	
Gender	Female (*n* = 29)	Male (*n* = 26)	Female (*n* = 42)	Male (*n* = 33)
Mean age (years)	37.1 ± 13.0	42.7 ± 13.6	34.2 ± 14.3	32.8 ± 13.6
Disease duration (years)	15.5 ± 9.5	12.6 ± 7.8	–	–
Extraintestinal manifestations	52%	38%	–	–
BMI	22.4 ± 4.8	23.1 ± 3.6	–	–

All data are presented as mean ± SD or %, respectively

*BMI* body mass index

### Age-related effects of CpG − 628 methylation rate and pressure pain threshold in healthy participants and Crohn patients

**Fig. 4:**
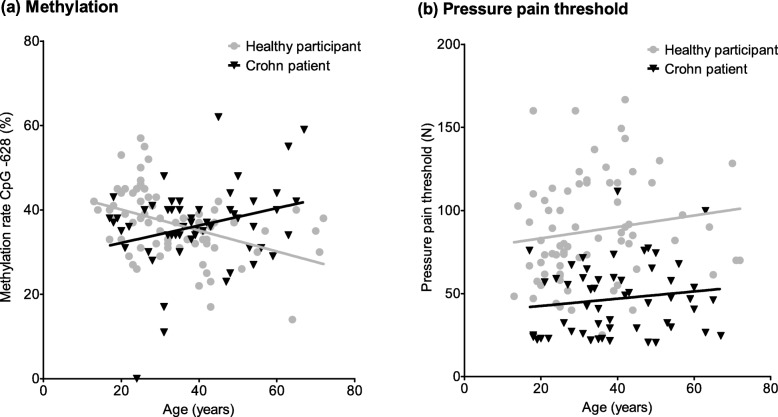
Age-related alterations in CpG − 628 methylation rate and pressure pain threshold. **a** In healthy participants, CpG − 628 methylation rate declines with increasing age. In contrast, it increases in Crohn patients. **b** PPT rises with increasing age in both cohorts

We next investigated age-related effects and found oppositional results for CpG − 628 methylation in healthy subjects and Crohn patients (Fig. 4a). Whereas in healthy subjects, the methylation rate decreases with age (*R*^2^ = 0.183), it increases in Crohn patients (*R*^2^ = 0.078). In contrast, PPTs seem to be unaffected by this circumstance (Fig. 4b, healthy participants *R*^2^ = 0.023, Crohn patients *R*^2^ = 0.019).

## Discussion

Several studies are dealing with genetic factors contributing to the susceptibility of IBD and additional environmental factors triggering the onset of the respective disease [44]. A familial aggregation has been observed [45], and 242 IBD risk loci have been identified so far [46], among them 38 displaying a shared genetic risk across populations [47]. In contrast to genetic factors, little is known about epigenetic alterations in Crohn’s disease. As reviewed by Moret-Tatay et al. [48], some microRNAs could possibly be used as biomarkers for Crohn’s disease in the blood of patients [49, 50]. Moreover, the DNA methylation pattern was shown to be altered in IBD. The promoter region of *TRIM39-RPP21*, which regulates the type I interferon pathway and is therefore important for viral immunity [51], is hypomethylated in patients with IBD [52]. In contrast, *TRAF6*, mediating signal transduction downstream of the tumor necrosis factor receptor superfamily and the interleukin-1 receptor/Toll-like receptor superfamily [53] is hypermethylated in IBD and seems to correspond to reduced mRNA expression [52].

In this study, we analyzed the *TRPA1* promoter methylation with regard to pain sensitivity. Evaluation of patient-controlled analgesia (PCA) and quantitative sensory testing (QST) of Crohn patients revealed significant differences between genders for heat pain threshold (HPT) and pressure pain threshold (PPT), with females showing higher sensitivity than males. Gender-biased pain thresholds have been indeed reported in several studies. As mentioned previously [42], gender effects on pain perception seem to be present after puberty in healthy individuals [54]. In young adults, females display higher pain sensitivity than males, regardless of their ethnicity [55]. Furthermore, females are more likely to suffer from pain conditions like chronic neuropathic pain [56]. A quantitative sensory study of adolescents revealed lower pressure pain thresholds and tolerance in individuals with chronic pain as compared to individuals without chronic pain; in general, females showed lower pain thresholds than males [57]. In human endotoxemia, females also display lower pain thresholds than males [58]. The intestinal permeability is increased in patients with Crohn’s disease, and the intestinal microflora is altered compared to healthy individuals [59]. Since bacterial lipopolysaccharides activate TRPA1 [60], defects in the barrier function of the intestinal epithelium could affect in a subsequent upregulation of TRPA1 expression. Indeed, *TRPA1* mRNA was found to be significantly upregulated in response to inflammation in mouse and human colon. The authors propose a protective role of TRPA1 since in a murine model for colitis the disease activity index and the histological score were lower in TRPA1 expressing mice than in the knockout mice after 10 days of dextran-sulfate treatment. This theory was further supported by the TRPA1-mediated downregulation of proinflammatory neuropeptides and cytokines under these serious inflammatory conditions [34]. However, the lower pain thresholds observed in females neither seem to display a Crohn’s disease-specific effect, nor a disease-specific effect in general. PPT differed significantly between both cohorts, with Crohn patients displaying lower thresholds than healthy participants. We partly used PPT data of the same subjects analyzed in previous studies [61, 62], which did not find differences in PPT between Crohn patients and healthy subjects. These contradictory outcomes are probably explained by the exclusion of subjects we performed due to missing data and after applying the criteria for data exclusion regarding the methylation values. Despite this fact, we analyzed these altered cohorts with regard to their methylation rates in the *TRPA1* promoter. Unfortunately, PPT data were the only QST data measured in healthy subjects. In both cohorts, the threshold is significantly lower for females than for males. Crohn patients may exhibit an altered regulation of pain perception, which can be caused by the inflammatory condition itself as well as by the medication Crohn patients receive. Reactive oxygen species, which are capable of activating TRPA1 [63], were shown to be produced in several gastrointestinal diseases, including Crohn’s disease [64]. In colorectal biopsies, a disease-related increase in reactive oxygen metabolites was detected [65, 66], as reviewed in [67]. Subsequent lipid peroxidation results in the formation of reactive carbonyl species like 4-hydroxynonenal and 4-oxononenal, which are able to activate TRPA1 as well [68, 69]. Regarding colonic distension, the rectal sensory threshold for pain is lower in children and adolescents with Crohn’s disease than in healthy controls [70]. On the other hand, in quiescent Crohn’s disease, the patients seem to display a visceral hyposensitivity and higher tolerance of rectal distension pressures [71]. However, our findings revealed lower pressure pain thresholds over the thenar muscles for Crohn patients compared to healthy subjects. When compared to healthy participants, only small or no alterations were found in methylation rates at the single CpG sites of the *TRPA1* promoter for Crohn patients. Due to this fact we concentrated further analyses on CpG − 628 since this CpG site showed a superordinate role regarding pain thresholds in healthy individuals in previous studies [41, 42]. We investigated this specific CpG site with respect to QST, PCA and other disease-associated parameters in Crohn patients. The comparison between the cohorts revealed differences in CpG − 628 methylation rate with respect to PPT. The higher the methylation rate, the lower is the PPT in both cohorts. Concerning gender, we detected differences between healthy participants and Crohn patients (Fig. 2). Whereas in the healthy volunteer group, linear regression showed no differences between males and females, Crohn patients showed a rather impressive effect in females as compared to males. Since *TRPA1* promoter methylation seems to have a higher influence on PPT in female than in male patients, these results might suggest that different pain treatments for female and male Crohn patients are reasonable and possible. Hence, broader studies should be carried out to verify the previous results in order to specify gender-dependent treatment options. Furthermore, CpG − 628 methylation rate was found to be a significant predictor not only for PPT but also for MPT. Similar to PPT, the effect of CpG − 628 methylation rate seems to also have a greater influence on females than on male patients for this parameter (Fig. 3). In addition, we detected a significant association between high CpG − 628 methylation rate and low BMI. This effect was identified as a gender-related side effect. However, we did not find a significant connection with other disease-associated parameters like disease duration or extraintestinal manifestations.

Aging is known to be a factor influencing the methylation status. The methylation rate can even be used to predict the age of different tissues and cell types [72]. We compared the methylation rate at CpG − 628 between both cohorts and found contrasting results. Whereas in healthy participants, the methylation rate decreases with advancing age, it increases in Crohn patients (Fig. 4a). This circumstance does not seem to have an influence on the PPT in any of the two cohorts, as PPT increases with advancing age (Fig. 4b). Therefore, the methylation mechanism of the *TRPA1* promoter seems to be dysregulated in Crohn patients, either as a cause or a result of the disease. Another factor could be the treatment with analgesic, immunosuppressive and other medication. Morphine was indeed shown to modify DNA methylation patterns [73], and glucocorticoids like dexamethasone may lead to an altered epigenetic regulation as well [74]. In patients suffering from rheumatoid arthritis, the drug methotrexate provokes changes in methylation rates [75, 76]. However, for the parameter disease duration, we could not detect a similar connection to CpG − 628 methylation (*R*^2^ = 0.001, data not shown).

A limitation of our study is the usage of DNA from whole blood cells for methylation analysis. It would have been more accurate to use TRPA1 expressing nociceptive neurons for methylation and expression analyses, but due to the inaccessibility of the tissue, we were limited to blood cells. Although in some cases variations in DNA methylation have been observed to be tissue-specific [77], in other cases, methylation patterns are similar in different tissues [78]. Alterations in the methylation pattern due to inflammation, smoking, consumption of alcohol and drugs, medication, as well as other factors which were not included in the study could have biased the results. Especially in patients suffering from chronic inflammatory disorders like Crohn’s disease, the treatment with immunomodulatory drugs over several years could have altered the methylation pattern. The large discrepancy in medication, disease duration and extraintestinal manifestations between the Crohn patients could have led to bias in the analyses. Unfortunately, PPT was the only QST measurement performed in Crohn patients and healthy controls, enabling a comparison of the two groups. Nevertheless, for the patient group, we were able to analyze several QST and PCA data with regard to methylation rate, and found CpG − 628 to be a significant predictor only for the dependent QST variables PPT and MPT. Therefore the remaining QST variables seem to be of minor relevance. Although in our study the number of cases analyzed was relatively low, it provided enough statistical power to reach significance level in statistical analyses.

## Conclusions

We found lower PPT for Crohn patients than for healthy participants and detected differences regarding the correlation between CpG − 628 methylation and PPT in the different genders, with a high correlation in female patients compared to the other groups. In Crohn patients, similar results were obtained for MPT, confirming the PPT results. Therefore further investigation of gender-dependent treatment options should be considered. Since we only found a significant effect regarding the correlation between CpG − 628 methylation and PPT in female patients, other mechanisms have to be involved in the altered pain perception in Crohn patients. Furthermore, *TRPA1* promoter methylation seems to be dysregulated in Crohn patients in an age-dependent manner.

## Methods

### Subjects

After approval by the local ethics committee and informed written consent from all volunteers, blood sampling of 75 healthy participants (42 females, 33 males) and 55 patients suffering from Crohn’s disease (29 females, 26 males) was conducted at the University of Erlangen-Nuremberg. In all patients, Crohn’s disease was histologically proved, they had no history of surgery within the last 3 months and did currently not take any pain medication. EDTA-blood was frozen at − 80 °C directly after sampling. Characteristics such as gender and age were documented for all subjects. Disease duration defined as years between diagnosis and blood sampling, extraintestinal manifestations, as well as BMI, were documented additionally for Crohn patients (Table 2). Samples were not matched for age.

### Patient-controlled analgesia

Blood samples were taken prior to surgery. The survey of patient-controlled analgesia (PCA) was conducted as described previously [62]. Postoperative morphine requirements were assessed in Crohn patients after major abdominal surgery. Patients received a fixed dose of nonsteroidal analgesic and were educated to administer the morphine themselves via a PCA device. Morphine consumption was documented for the first 72 h after surgery. The consumption of morphine was normalized to body weight and time.

### Quantitative sensory testing

Somatosensory profiles in Crohn patients were estimated via quantitative sensory testing (QST) as described previously [62], according to the protocol of the German Research Network on Neuropathic Pain [79, 80]. Stimuli, which were applied to the dorsum of both hands, included the following thermal and mechanical tests: to perform thermal sensory testing a TSA 2001-II thermal sensory analyzer (Medoc Ltd., Israel) was used. Baseline temperature was 32 °C and the stimulus was gradually increased about 1 °C/s until the subject pressed a break-off button to determine the specific thermal threshold for cold detection (CDT), warm detection (WDT), cold pain (CPT), or heat pain (HPT). Temperature thresholds were assessed as the difference from the baseline value. Cutoff temperatures were 0 and 50 °C. Mechanical detection thresholds (MDT) were measured using modified von Frey filaments with forces between 0.25 and 512 mN (Opti Hair, Marstock nervtest, Germany), whereas mechanical pain thresholds (MPT) were determined using a set of seven custom-made weighted pinprick stimulators with fixed stimulus intensities between 8 and 512 mN (Department of Physiology and Pathophysiology, Mainz, Germany)—the final threshold for both tests was the geometric mean of 5 series of ascending and descending stimuli [81]. In order to determine mechanical pain sensitivity (MPS) as a stimulus-response-function, pinprick stimulators were also used. Subjects had to rate pain on a numerical scale from 0 (no pain) to 100 (strongest imaginable pain) while pinpricks were applied five times in random order. MPS was then computed as the mean of pain rating after the pinprick stimuli. The wind-up ratio (WUR) was calculated as the mean of five series of 10 repetitive pinprick stimuli (256 mN, 1/s), divided by the mean rating after five single stimuli [82]. Over the processus styloideus ulnae determination of vibration detection threshold (VDT) was performed using a Rydel-Seiffer tuning fork (64 Hz, 8/8 scale) until the subject could not feel vibration anymore. VDT was calculated as the mean of a disappearance threshold with three stimulus repetitions. Pressure pain thresholds (PPT) of healthy participants and Crohn patients were measured by an algometer (FDN 200, Wagner Instruments, Greenwich, CT, USA) with an area of contact of 1 cm^2^ over the thenar muscles. PPT was determined with 3 series of ascending stimulus intensities until the subjects expressed pain. Mean values were used for statistical analyses.

### DNA isolation and bisulfite reaction

DNA for methylation analysis of both cohorts was extracted from blood. The cleanup of genomic DNA from healthy participants was performed using the Nucleo-Mag® Blood 200 μl DNA Kit (Macherey-Nagel, Düren, Germany). Genomic DNA from Crohn patients was extracted according to standard procedures with an automated chemagic MSM I system (Perkin Elmer, Baesweiler, Germany). Bisulfite conversion and DNA purification were conducted via the EpiTect® 96 Bisulfite Kit (QIAGEN, Hilden, Germany). DNA concentrations were determined via a Nanodrop 1000 spectrophotometer (VWR, Radnor, PA, USA). A Biomek® NxP (Beckman Coulter, Brea, CA, USA) was used for pipetting, transferring and purification steps.

### Amplification of target sequences

Amplification of *TRPA1* promoter target sequences of the purified bisulfite-converted DNA was conducted in 2 different fragments, as described previously [42]. The first promoter fragment of *TRPA1* was amplified using the forward primer 5′-GTTTGTATTAGATAGTTTTTTTGTTTG-3′ and the reverse primer 5′-TCCTACAAACCTATATTTCCCAC-3′, the second fragment via the forward primer 5′-GGGGTAGGGTAAGGGGTTTT-3′ and the reverse primer 5′-TACACACACCCCAAAACTTACAAC-3′, using touchdown PCRs [83] with starting temperatures of 65 °C. Oligonucleotides applied as primers were ordered from Metabion (Steinkirchen, Germany), PCRs were performed on a C1000 TM Thermal Cycler (BIO-RAD, Hercules, CA, USA) using the HotStarTaq® Master Mix Kit (QIAGEN, Hilden, Germany).

### Sequencing

Amplification products of touchdown PCRs were purified using the Agencourt® AMPure® XP magnetic beads (Beckman Coulter, Brea, CA, USA). Sequencing PCRs of the target fragments were performed using the Big-Dye® Terminator v3.1 Cycle Sequencing Kit (Applied Biosystems, Foster City, CA, USA). Oligonucleotides used for sequencing PCR were 5′-GTTTGTATTAGATAGTTTTTTTGTTTG-3′ (first fragment) and 5′-CTACCCCCAAAAAAACCTCCAAC-3′ (second fragment). For amplification of the first fragment, cycling conditions apply as follows: 1 min 96 °C, 28 × (10 s 96 °C, 5 s 50 °C, 4 min 60 °C). For the second fragment, cycling conditions for AT-rich sequences were applied according to manufacturer recommendations: 1 min 96 °C, 26 x (5 s 96 °C, 90 s 60 °C, 90 s 50 °C). The amplified fragments covered 55 CpG sites within the promoter region of the *TRPA1* gene (from − 734 bp to + 335 bp of the first exon). Products of the sequencing PCR were purified using Agencourt CleanSeq® XP magnetic beads (Beckman Coulter, Brea, CA, USA), afterward sequencing was performed on a 3500XL genetic analyzer from ABI Life Technologies (Grand Island, NY, USA) according to the manufacturer’s instructions.

### Determination of methylation rates

Sequence analysis and determination of methylation rates for each CpG site were conducted using the Epigenetic Sequencing Methylation (ESME) analysis software [84]. The methylation rate of each CpG site per subject was estimated by the ratio between normalized peak values of cytosine and thymine.

### Statistical analyses

**Fig. 5:**
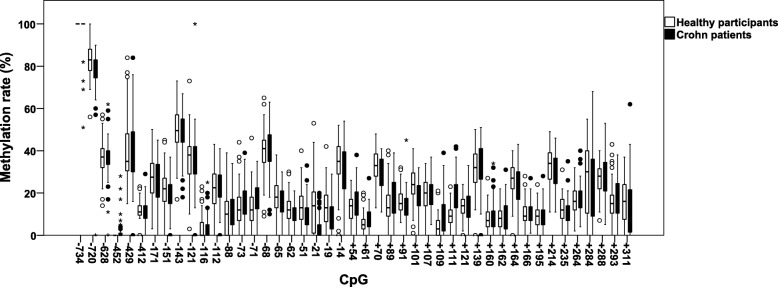
Overview of methylation rates for single CpGs. Methylation rates are denoted from 0 up to 100% methylation for each CpG. Methylation rates between different CpGs show substantial variation. Only slight differences between healthy participants and Crohn patients are distinguishable at some CpG positions. Boxes were generated for 50% of the occurrence, outliers with values between 1.5 and 3 box lengths distance are depicted as circles, extremes with more than 3 box lengths distance are represented as stars

Sequence quality was assessed via Sequence Scanner v1.0 software (ABI Life Technologies, Grand Island, NY, USA). Only sequences with a Quality Value > 20 for Trace Score in the Quality Control Report were included for further analyses (11 of 147 sequences were excluded due to low quality). All statistical calculations were performed using the Statistical Package for the Social Sciences (SPSS 24, IBM, Armonk, NY, USA). We used GraphPad Prism for Windows 5.03 for data illustration (Graphpad Software Inc., La Jolla, CA, USA). Single CpGs with less than 95% sequencing success among the samples were excluded, which applied to CpG − 161, − 40, and − 38 ahead of the first exon, as well as CpG + 147, + 333, and + 335 of the first exon. Samples with less than 95% sequencing success of overall CpGs, which applied to 6 samples, and CpG sites with less than 5% inter-individual variability, which applied to CpG − 480, + 13, + 15, and + 191 were also not further analyzed. After applying the criteria for data exclusion as mentioned previously, the remaining number of samples dropped from 147 (88 healthy participants, 59 Crohn patients) to 130 (75 healthy participants, 55 Crohn patients), the number of analyzed CpGs dropped from 55 to 45. Methylation levels for individual CpG sites are provided in Fig. 5. No apparent differences in methylation rate at single CpG sites between healthy participants and Crohn patients were visible in this figure at first sight. Deviance from normal distribution was checked according to Shapiro-Wilk. In case of normally distributed variables, parametric methods were used; for all other cases, nonparametric tests were applied. Multiple linear regression (BACKWARD method) was conducted to identify significant predictors for values of PCA, QST, and demographic and disease-associated data. In each analysis, a *p* value of < 0.05 was considered significant.

## Data Availability

The datasets used and/or analyzed during the current study are available from the corresponding author on reasonable request.

## Abbreviations

CDT: Cold detection threshold
CGRP: Calcitonin gene-related peptide
CPT: Cold pain threshold
HPT: Heat pain threshold
IBD: Inflammatory bowel disease
MDT: Mechanical detection threshold
MPS: Mechanical pain sensitivity
MPT: Mechanical pain threshold
PCA: Patient-controlled analgesia
PPT: Pressure pain threshold
QST: Quantitative sensory testing
SNP: Single-nucleotide polymorphism
SP: Substance P
TNBS: 2,4,6-trinitrobenzene-sulfonic-acid
TRP: Transient receptor potential
TRPA1: Transient receptor potential ankyrin 1
VDT: Vibration detection threshold
WDT: Warm detection threshold
WUR: Wind-up ratio
