# Correction to: Transient receptor potential ankyrin 1 promoter methylation and peripheral pain sensitivity in Crohn’s disease

**DOI:** 10.1186/s13148-021-01059-9

**Published:** 2021-03-30

**Authors:** Sara Gombert, Mathias Rhein, Andreas Winterpacht, Tino Münster, Thomas Hillemacher, Andreas Leffler, Helge Frieling

**Affiliations:** 1 Laboratory for Molecular Neuroscience, Department of Psychiatry, Socialpsychiatry and Psychotherapy, Hannover Medical School, Feodor-Lynen-Str. 35, 30625 Hannover, Germany; 2 Institute of Human Genetics, Friedrich-Alexander University Erlangen-Nuremberg, Erlangen, Germany; 3 Department of Anesthesiology, Friedrich-Alexander University Erlangen-Nuremberg, Erlangen, Germany; 4 Clinic for Anesthesiology and Critical Care, Hospital of the Order of St.John of God Regensburg, Regensburg, Germany; 5 Department of Psychiatry and Psychotherapy, Paracelsus Medical University Nuremberg, Nuremberg, Germany; 6 Department of Anesthesiology and Intensive Care Medicine, Hannover Medical School, Hannover, Germany

## Correction to: Clinical Epigenetics (2020) 12:1 https://doi.org/10.1186/s13148-019-0796-9

Following publication of the original article [1], an error was identified in the use of reference 42 which was cited insufficiently. Data of healthy participants, which were used for comparison with the Crohn patient group in Figures 1, 2, 4 and 5, and Table 2, were already analyzed and published previously in:

42. Gombert S, Rhein M, Eberhardt M, Munster T, Bleich S, Leffler A, et al. Epigenetic divergence in the TRPA1 promoter correlates with pressure pain thresholds in healthy individuals. Pain. 2017;158(4):698–704. Epub 2016/12/29.
